# Supplementary material for: Exploring protein hotspots by optimized fragment pharmacophores
Source: Nat Commun. 2021 May 27;12:3201. doi: 10.1038/s41467-021-23443-y (PMC8159961; doi:10.1038/s41467-021-23443-y)
Supplement: Supplementary file 1 — Supplementary information [file 41467_2021_23443_MOESM1_ESM.pdf]

# Supplementary information

## Exploring protein hotspots by optimized fragment pharmacophores

Dávid Bajusz[1], Warren S. Wade[2], Grzegorz Satała[3], Andrzej J. Bojarski[3], Janez Ilaš[4], Jessica Ebner[5], Florian Grebien[5], Henrietta Papp[6], Ferenc Jakab[6], Alice Douangamath[7], Daren Fearon[7], Frank von Delft[7], Marion Schuller[8], Ivan Ahel[8], Amanda Wakefield[9,10], Sándor Vajda[9,10], János Gerencsér[2], Péter Pallai[2], György M. Keserű[1,*]

[1] *Medicinal Chemistry Research Group, Research Centre for Natural Sciences, Magyar tudósok krt. 2, 1117 Budapest, Hungary*

[2] *BioBlocks, Inc., 9885 Mesa Rim Road, Suite 101, San Diego, CA 92121*

[3] *Maj Institute of Pharmacology Polish Academy of Sciences, 12 Smetna Street, 31-343 Kraków, Poland*

[4] *University of Ljubljana, Faculty of Pharmacy, Aškerčeva cesta 7, 1000 Ljubljana, Slovenia*

[5] *Institute for Medical Biochemistry, University of Veterinary Medicine, Veterinaerplatz 1, 1220 Vienna, Austria*

[6] *National Laboratory of Virology, Szentágothai Research Centre, University of Pécs, Ifjúság útja 20., 7624, Pécs, Hungary*

[7] *Diamond Light Source Ltd., Harwell Science and Innovation Campus, Didcot OX11 0QX, UK*

[8] *Sir William Dunn School of Pathology, University of Oxford, Oxford OX1 3RE, United Kingdom*

[9] *Department of Chemistry, Boston University, Boston, MA, 02215, USA*

[10] *Department of Biomedical Engineering, Boston University, Boston, MA, 02215, USA*

* Corresponding author: György M. Keserű, email: keseru.gyorgy@ttk.hu

**Table of contents**

1. Clustering of experimental structures to non-redundant set of binding pharmacophores...... 3

1.1 Input dataset preparation ........................................................................................ 3

1.2 E-Pharmacophore generation .................................................................................. 3

1.3 Hot-spot detection.................................................................................................... 4

1.4 Clustering of E-pharmacophore hypotheses ............................................................ 5

1.5 Detection of submodels ........................................................................................... 6

1.6 Occurrence of pharmacophoric features in the non-redundant set of binding pharmacophores ........................................................................................................ 6

1.7 Pharmacophoric features and their nomenclature .......... 7
2. Optimization of the SpotXplorer pilot library .......... 8
2.1 Ligand preparation and screening .......... 8
2.2 Library optimization .......... 8
3. Top twenty most populated pharmacophore models .......... 9
4. Pharmacophore enrichment plots for SpotXplorer 0 and commercial fragment collections 11
5. *In vitro* screening for selected GPCRs .......... 13
5.1 Cell culture and preparation of cell membranes for radioligand binding assays .......... 13
5.2 Radioligand binding assays .......... 13
6. Protease inhibitory assays .......... 13
7. SETD2 Chemiluminescence assay .......... 14
8. Cell viability assay .......... 15
9. X-ray fragment screening against COVID-19 targets .......... 15
10. *In vitro* SARS-CoV-2 antiviral activity screen .......... 17
11. Supplementary references .......... 18

## 1. Clustering of experimental structures to non-redundant set of binding pharmacophores

*1.1 Input dataset preparation*

A subset of the experimental structures deposited in the Protein Data Bank was downloaded to constitute the basis of our analysis. The subset was defined so that each structure has met the following criteria:

i) The structure has a ligand.

ii) The type of the macromolecule is protein (excluding protein-DNA and protein-RNA complexes).

iii) The experimental method is X-Ray crystallography, with a resolution of 2.5Å or better.

The structures were further filtered based on their ligands. In particular, an sdf file containing the first occurrence of each ligand in each PDB entry (a total of 144760 occurrences of 15523 ligands) was downloaded and processed. This set was first focused to ligands with 10-16 heavy atoms (following the fragment definition of Murray and Rees[1]) to yield a total of 13611 occurrences of 2905 unique ligands. The further filtering of this set involved three steps:

i) Ligands with ligand types other than "non-polymer" (such as "D-saccharide", "L-peptide linking", etc.) were filtered out.

ii) Ligands containing zero rings were excluded.

iii) The occurrences of each of the ligands were counted and those with more than ten occurrences were manually curated to exclude buffers, surfactants, hydrocarbons, and a few other, exotic substances (e.g. an iron-sulfur cluster).

The complete list of ligands that were filtered out in step iii) is provided in the **Supplementary Data** file (with PDB Ligand IDs and SMILES strings). The resulting dataset contained a total of 3944 occurrences of 1859 ligands, in a total of 3827 PDB entries. (The complete list of PDB entries is provided in the **Supplementary Data** file.) For dataset processing, the KNIME[2] and Cinfony[3] cheminformatics toolkits were used.

*1.2 E-Pharmacophore generation*

The 3827 PDB entries were further processed in the following manner:

i) Each PDB file was split, to a total of 7858 individual chains.

ii) The resulting chains were checked, and those that did not contain any of the 1859 ligands were removed, leaving a total of 6738 single-chain PDB files.

iii) The protein structures were prepared with Schrödinger's Protein Preparation Wizard.[4] Briefly, hydrogens were added, protonation states and hydrogen bond orientations were optimized (PROPKA), missing side chains were modeled (Prime) and a short restrained minimization was run (Impref, $RMSD_{heavy\ atoms} \leq 0.30$ Å). Since some of the preparation jobs have failed due to various errors, a total of 6415 chains of 3666 PDB entries have been successfully prepared.

iv) Some PDB entries contained covalently bound ligands. These were omitted, leaving a total of 6106 chains in 3503 PDB entries. Grid generation was successful for 5965 chains in 3422 entries.

v) Next, Glide XP docking was utilized to score the co-crystallized ligands in place, and generate XP descriptors (necessary for E-pharmacophore generation). The generation of XP descriptors entails a detailed breakdown of the GlideScore to score contributions from the different moieties (atoms, rings) in the molecule.[5] Docking was successful for 5953/3413 chains/PDB entries.

vi) E-pharmacophore generation (in single ligand mode) involves an analysis of the XP descriptors and building a pharmacophore model based on the pharmacophore features with the greatest GlideScore contributions.[6] This step can be thought of as selecting those features from the molecule that contribute to ligand binding the most effectively. E-pharmacophore generation was successful for 5795/3343 chains/PDB entries. The maximum number of pharmacophore features to be included in the final model was 4 (contrary to our effort for optimizing the SpotXplorer pilot library for 2- and 3-point pharmacophores), for the following reasons:

a) This way, 3-point pharmacophores correspond to cases when there were actually no further interactions with a significant contribution to protein-ligand binding (instead of being artificially enforced to select the three best among more features).

b) Limiting the number of features to four agrees with the concept of binding pharmacophores as the smallest set of key pharmacophoric features, with the largest contributions to fragment binding.[1]

c) Having the set of 4-point pharmacophores in hand enables the design of larger fragment libraries by optimizing for 4-point pharmacophores, in addition to 2- and 3-point ones.

*1.3 Hot-spot detection*

We used FTMap,[7,8] a computational method for determining binding hot spots. FTMap is a computational analog of fragment screening experiments by X-ray crystallography[9,10] or NMR.[11] The method distributes small organic probe molecules of varying size, shape, and polarity on a dense grid defined on the macromolecule surface, finds the most favorable positions for each probe type, performs local energy minimization allowing for probe flexibility, then clusters the probes and ranks the clusters on the basis of their average energy. The FTMap server currently uses 16 organic molecules as probes (ethanol, isopropanol, isobutanol, acetone, acetaldehyde, dimethyl ether, cyclohexane, ethane, acetonitrile, urea, methylamine, phenol, benzaldehyde, benzene, acetamide, and N,N-dimethylformamide). For each probe type, the initial sampling uses the fast Fourier transform (FFT) correlation algorithm to sample billions of positions on translational and rotational grids, consisting of 0.8 Å translations and of 500 rotations at each location. Probe positions are scored using a detailed energy expression that includes attractive and repulsive van der Waals terms, electrostatic interaction energy based on Poisson-Boltzmann calculations, a cavity term, and a structure-based pairwise interaction potential. The 2000 lowest energy poses for each probe are retained and energy minimized using the CHARMM potential[12] with the Analytic Continuum Electrostatic (ACE) model[13] to account for electrostatics and solvation, allowing for probe flexibility. For each probe type, the minimized poses are clustered with a 4Å radius, starting

with the lowest energy structure. The clusters are ranked on the basis of their Boltzmann averaged energies. The low energy clusters of different probes are then clustered, and regions that bind multiple probe clusters are defined as the predicted binding hot spots. The hot spots are ranked based on the number of different probe clusters they bind. It was shown that any site capable of binding small molecules with micromolar or better affinity requires the binding of at least 16 probe clusters, which can be considered a simple condition for druggability.[14] The FTMap server has been extensively tested in a variety of applications, including for fragment based drug discovery,[15,16] and is available at http://ftmap.bu.edu.

Here, we performed FTMap analysis on the protein-fragment complexes and considered only fragments bound to the three highest ranked hotspots. For each of the fragment-bound structures, we mapped an individual chain with FTMap. We then compared the location of the top three FTMap hotspots to the location of the fragment. If the fragment was located within 3 Å of a top ranked hotspot, we considered the fragment to be bound to a hotspot in the protein. This approach reflects on evolutionary selection, since there is a limited number of endogenous ligands, targeting a limited number of binding sites.

*1.4 Clustering of E-pharmacophore hypotheses*

The resulting pharmacophore hypotheses were clustered in a stepwise manner, in two steps.

i) The models were first clustered according to the pharmacophore feature set, which was encoded with letters (A – acceptor, D – donor, H – hydrophobic, N – negative ionizable, P – positive ionizable, R – aromatic ring) and sorted alphabetically, i.e. a model containing two acceptors and an aromatic ring is denoted with AAR. We have termed these level 1 clusters for the sake of convenience.

ii) The resulting level 1 clusters were further clustered based on a spatial alignment of the pharmacophore models. To that end, a complete RMS (root-mean-squared) distance matrix was calculated for each level 1 cluster. The distance matrix contains pairwise RMSD values between each pairs of pharmacophore models in a level 1 cluster, calculated according to the following:

a) Each model is preprocessed, so that its center coincides with the origin, [0,0,0]. Then, each pair of models is fitted to each other: one model is kept fixed, while the other is rotated around and translated along the x, y and z axes (a total of six variables), until the RMSD value reaches a minimum. The minimization is carried out with the Powell algorithm, as implemented in the scipy package.[17] For each pair of models *A* and *B*, two such calculations are executed: one with *A* as the fixed model, and one with *B*, with the final output being the smaller RMSD value.

b) During the RMSD calculation, the equivalence between features of the same type is accounted for with the Hungarian algorithm[18] (*e.g.* in the case of two models with three acceptors each, the features are assigned to each other in such a way, that the sum of their pairwise distances is the lowest).

On the resulting distance matrix, a hierarchical agglomerative clustering is carried out with the Euclidean distance metric and complete linkage. An RMSD cutoff of 2.0 Å is applied to assign level 2 clusters, which are labeled as AAR_0, AAR_1, etc. Since the linkage method is complete, the RMS distance of any two pharmacophore models in the same cluster is less than or equal to 2.0 Å. For each level 2 cluster, the pharmacophore model closest to the cluster

centroid is identified. The total number of clusters resulting from this process was 141 level 1 clusters (excluding 6, which contain one feature in each) and 425 level 2 clusters. From the level 2 clusters, 33 are 2-point, 84 are 3-point and 308 are 4-point pharmacophores. The cluster centroids constitute the non-redundant set of fragment binding pharmacophores, and are used later for the pharmacophore screening of fragment libraries.

### *1.5 Detection of submodels*

As mentioned in the main text, there are many cases when a smaller pharmacophore model contains a subset of the features of a larger model and can be fitted onto this subset in 3D. In such cases, the smaller model is said to be a submodel of the larger one. To account for such relationships, each pharmacophore model in the non-redundant set was spatially fitted to all of the larger pharmacophores that contained all of its features, in the same way as detailed above (accounting for equivalent features). Whenever a pair of pharmacophores with an RMSD less than or equal to 2.0 Å was found, the smaller was flagged as a submodel of the larger one, ultimately generating a list of submodels for each 3- and 4-point pharmacophore. The list was used for post-processing the results of pharmacophore screenings: if a molecule was reported as a hit for a larger pharmacophore, it was set as a "non-hit" for all of its submodels. This ensures that in the pharmacophore-optimized fragment library, smaller pharmacophores are represented on their own right, rather than then trivially being present in molecules with larger pharmacophores.

### *1.6 Occurrence of pharmacophoric features in the non-redundant set of binding pharmacophores*

In the table below, we have compiled the percent occurrences of a certain number (0,1,2…) of specific features (A, D, etc.) and more general feature types (H-bond, ionic centers, etc.) in the 2/3/4-pt. pharmacophores of the non-redundant set (425). Notably, 42% of 2-pt. pharmacophores does not contain any directional (H-bond) features: this drops to 24% and 5% for 3-pt. and 4-pt. pharmacophores, respectively (12% for the total set of pharmacophores). If we also account for other polar features (negative or positive ionic centers), then there are only 18/6/1% of 2/3/4-pt. pharmacophores that do not contain any of these (3% for the total set of pharmacophores). This is in line with our previous observation that apolar desolvation of fragments alone cannot compensate for rigid-body entropy loss upon binding (15-20 kJ/mol, *vs.* maximum 1 kJ/mol per heavy atom gained by desolvation).[19] By contrast, 41% of the pharmacophores do not contain any apolar features (hydrophobic moieties or aromatic rings). These observations point to the conclusion that in order to ensure higher affinity binding, the hot spot must provide specific polar interactions, resulting in the binding of fragments to be typically enthalpy-driven.[19]

**Supplementary Table 1. Percent occurrences of pharmacophore features in the non-redundant pharmacophore set.**

| % | # | A | D | H | N | P | R | Hbond (A,D) | ion (N,P) | polar (A,D,N,P) | apolar (H,R) |
|---|---|---|---|---|---|---|---|---|---|---|---|
| 2-pt. | 0 | 64 | 70 | 76 | 70 | 88 | 67 | 42 | 58 | 18 | 48 |
| | 1 | 30 | 24 | 18 | 21 | 12 | 27 | 36 | 33 | 33 | 33 |

| | | | | | | | | | | | |
|---|---|---|---|---|---|---|---|---|---|---|---|
| | 2 | 6 | 6 | 6 | 9 | 0 | 6 | 21 | 9 | 48 | 18 |
| 3-pt. | 0 | 51 | 54 | 69 | 61 | 83 | 39 | 24 | 46 | 6 | 24 |
| | 1 | 37 | 33 | 23 | 33 | 17 | 45 | 40 | 45 | 30 | 40 |
| | 2 | 12 | 13 | 7 | 6 | 0 | 14 | 27 | 8 | 40 | 30 |
| | 3 | 0 | 0 | 1 | 0 | 0 | 1 | 8 | 0 | 24 | 6 |
| 4-pt. | 0 | 25 | 26 | 75 | 62 | 83 | 59 | 5 | 48 | 1 | 45 |
| | 1 | 35 | 38 | 18 | 28 | 16 | 31 | 14 | 40 | 7 | 31 |
| | 2 | 28 | 27 | 5 | 9 | 1 | 9 | 30 | 12 | 16 | 16 |
| | 3 | 10 | 7 | 2 | 0 | 0 | 1 | 31 | 0 | 31 | 7 |
| | 4 | 2 | 1 | 1 | 0 | 0 | 0 | 20 | 0 | 45 | 1 |
| all | 0 | 33 | 35 | 74 | 63 | 84 | 56 | 12 | 48 | 3 | 41 |
| | 1 | 35 | 36 | 19 | 29 | 16 | 33 | 21 | 40 | 14 | 33 |
| | 2 | 23 | 23 | 6 | 9 | 0 | 10 | 29 | 11 | 23 | 19 |
| | 3 | 7 | 5 | 2 | 0 | 0 | 1 | 24 | 0 | 27 | 7 |
| | 4 | 1 | 1 | 0 | 0 | 0 | 0 | 14 | 0 | 33 | 1 |

*1.7 Pharmacophoric features and their nomenclature*

Schrödinger's pharmacophore modelling software (Phase) implements six types of pharmacophoric features: A – H-bond acceptor, D – H-bond donor, H – hydrophobic group, N – negative charge, P – positive charge, R – aromatic ring.[20,21] A spatial arrangement of two or more pharmacophoric features constitutes a pharmacophore model: in this work, these are commonly referred to simply as "pharmacophores" (or "binding pharmacophores"), optionally specifying the number of constituting features, e.g. 3-point pharmacophores. A pharmacophore is labelled by its constituting features in alphabetical order and an arbitrarily assigned number that identifies the specific spatial arrangement of this feature set, e.g. NNR_0. A pharmacophore can contain more features of the same type, see e.g. the NNR_0 pharmacophore below in section 3, where the two N-s correspond to two distinct negative ionic centers that likewise have to be matched by two distinct negatively charged functional groups during screening. Also, it is important to note that certain functional groups (e.g. hydroxyl groups) can – but do not need to! – correspond to more than one pharmacophoric feature. For example, the hydroxyl group in ADR_0 (see section 3) contributes significantly to ligand binding (as evaluated by the Glide XP docking score contributions) as both an acceptor and a donor. To match these two features, a group with an equally close acceptor-donor pair (such as OH, SH or $NH_2$) must be present. On the other hand, the hydroxyl group in AR_0 contributes only as an acceptor to ligand binding, therefore it can be matched by groups that only act as acceptors (e.g. OMe, pyridine-type aromatic nitrogen, etc.).

## 2. Optimization of the SpotXplorer pilot library

### *2.1 Ligand preparation and screening*

Bioblocks and other vendor available fragment sets were filtered for high quality fragment properties with slightly relaxed cutoffs to allow more picking options:

i) Number of heavy atoms between 7 and 17
ii) MW between 100 and 250 (or with 1 Br 280)
iii) Number of Rings between 1 and 3
iv) ≤ 4 diastereomers
v) ≤ 1spiro ring junction
vi) ≤ 2 bridgehead atoms
vii) At least 1 heteroatom present per counted ring to remove R or H only fragments
viii) ≤ 3 hydrogen bond donors
ix) ≤ 8 hydrogen bond acceptors
x) ≤ 3 rotatable bonds, excluding methoxy
xi) ≤ 1 total Cl and Br atoms
xii) ≤ 1 S atom
xiii) No reactive functional groups or PAINs[22]

The filtered fragment sets were prepared with the Schrödinger software suite.[4] Ligand preparation entailed the generation of ligand protomers, conformers, etc.*;* its parameters were optimized by reviewing several test cases by three medicinal chemists. After checking a large number of possible settings, we have settled on the following protocol:

i) Protomers and tautomers are generated with Epik, in a pH range of 7.4 ± 1.5, after removing salts.[23,24] Stereoisomers were retained where specified (maximum 32 combinations were generated for non-specified stereo-centers).

ii) A full conformational search was conducted with Macromodel, with a mixed torsional/low-mode sampling protocol (with enhanced torsional sampling), max. 200 steps (max. 20 per rotatable bond), an energy window of 15 kJ/mol and an RMSD cutoff of 1.0 Å (other settings were kept as their default values).[25] Consequently, there was no fixed number of conformations: for each fragment, all conformations were kept that were no more than 15 kJ/mol above the "global minimum" (the lowest-energy conformation that was actually identified by Macromodel) and deviated by more than 1.0 Å in RMSD from all previously identified conformations. Since all of these are (relatively) low-energy conformers, one matching conformer was considered to be enough during screening.

The prepared fragment libraries were converted to Phase databases and screened against the 425 non-redundant binding pharmacophores with Phase.[20,21]

### *2.2 Library optimization*

In the SpotXplorer approach, library optimization includes two steps. First, the desired number of molecules are selected by the MaxMin algorithm,[26] based on the distances of their pharmacophore fingerprints (see main text, Figure 1C for illustration). Specifically:

i) The first molecule is selected randomly.

ii) All pairwise distances between the set of selected molecules and the rest of the molecules in the available pool is calculated. For each molecule in the pool, the minimum distance (to any of the selected molecules) is kept.

iii) The pool molecule with the largest such minimum-distance is included to the set of selected molecules and removed from the pool. The algorithm returns to step ii).

iv) If the desired number of molecules is selected, the algorithm halts.

Then, the second part of the algorithm can swap a selected molecule to a pool molecule if the swap increases the objective function of the algorithm, which is the sum of:

i) compound diversity: mean pairwise distance of the pharmacophore fingerprints of the selected molecules

ii) pharmacophore diversity: the column-wise analog of compound diversity, i.e. mean pairwise distance of the "compound fingerprints" of the pharmacophores (the objective being that each pharmacophore is represented by a different set of compounds)

iii) pharmacophore coverage: ratio of pharmacophores with at least one matching molecule.

For compiling the SpotXplorer 0 library, the 2- and 3-point pharmacophores were optimized. The algorithm utilizes functions implemented in the scipy package, enabling the use of several distance measures (here, the Euclidean distance was applied).[17]

### 3. Top twenty most populated pharmacophore models

To demonstrate the power of two-level clustering, here we include a brief analysis of the twenty most "popular" pharmacophores, in terms of the number of PDB entries and unique protein targets (identified by Uniprot IDs) in which they appear.

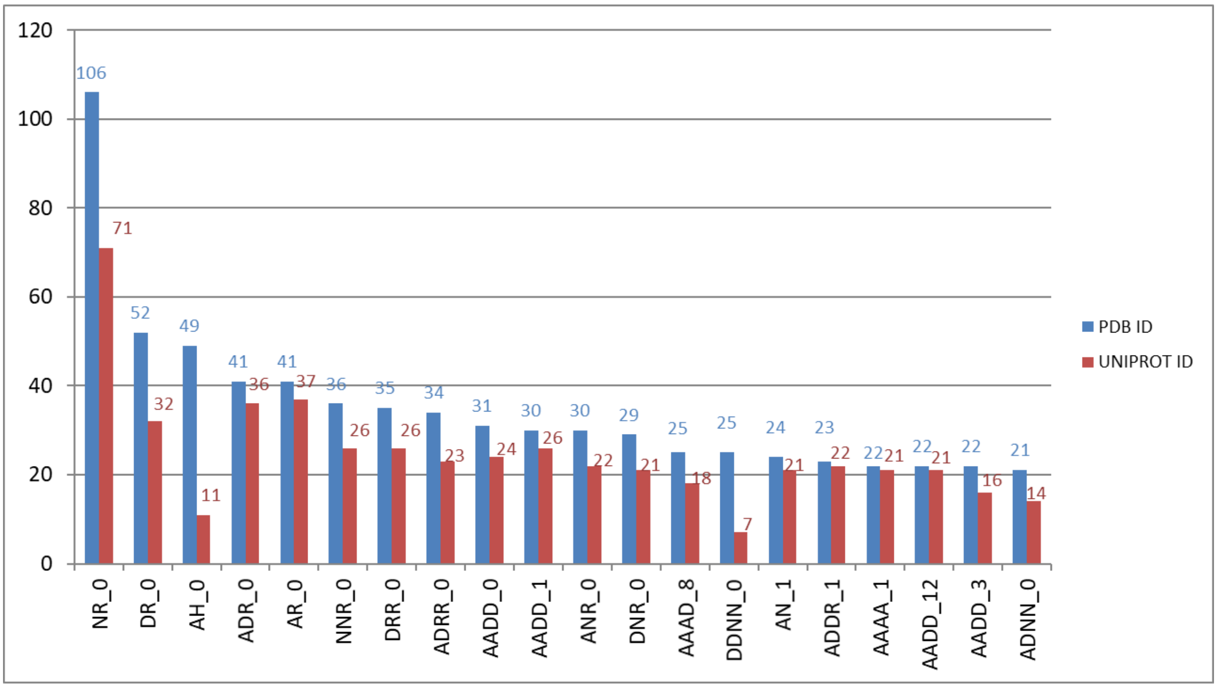


**Supplementary Figure 1. Number of PDB entries and unique protein targets for the twenty most populated pharmacophores.**

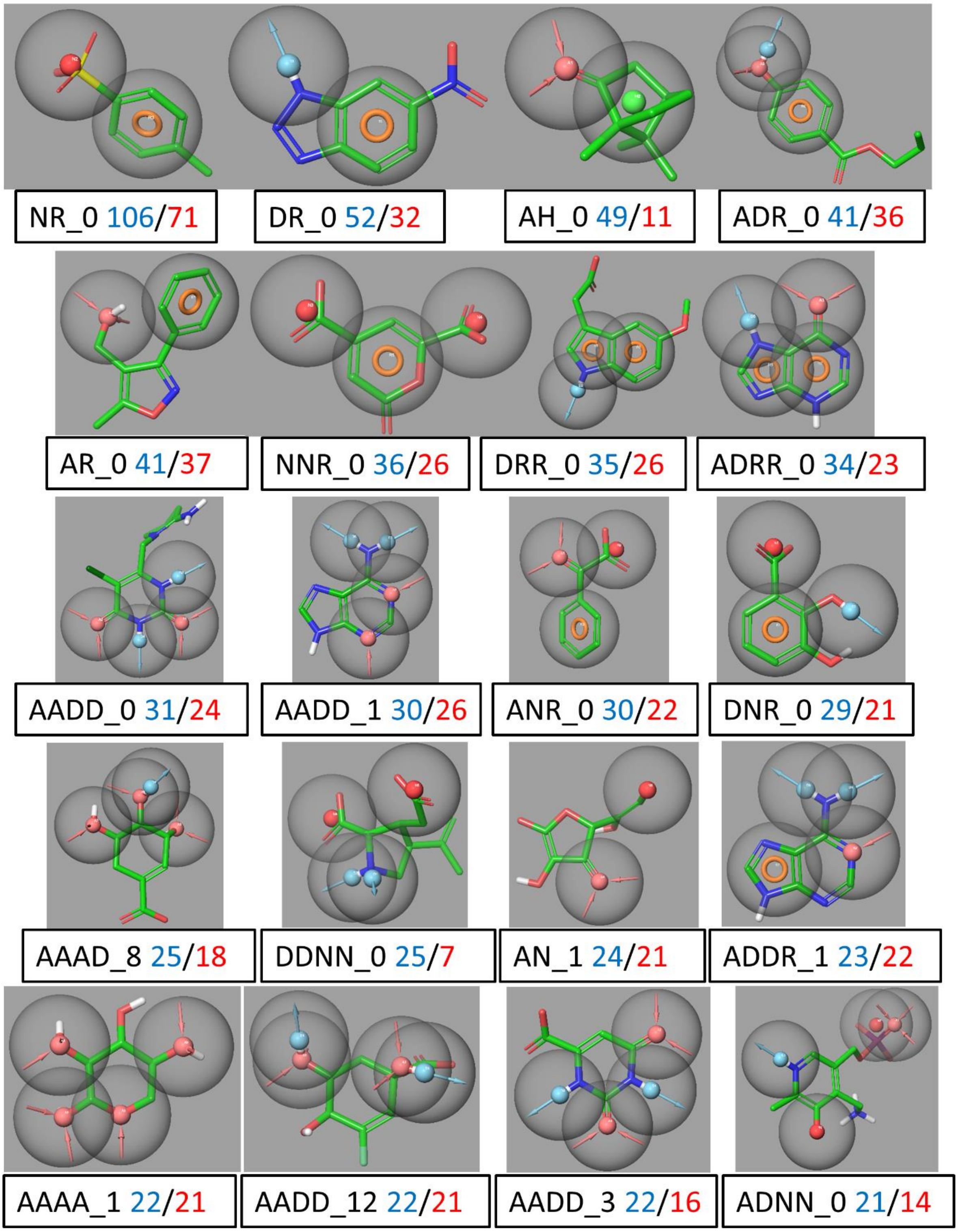


**Supplementary Figure 2. Top twenty most populated pharmacophore models.** The number of unique PDB entries and unique Uniprot entries (i.e. unique protein targets) are indicated in blue and red, respectively. The ligand shown corresponds to the structure closest to the cluster centroid.

**4. Pharmacophore enrichment plots for SpotXplorer 0 and commercial fragment collections**

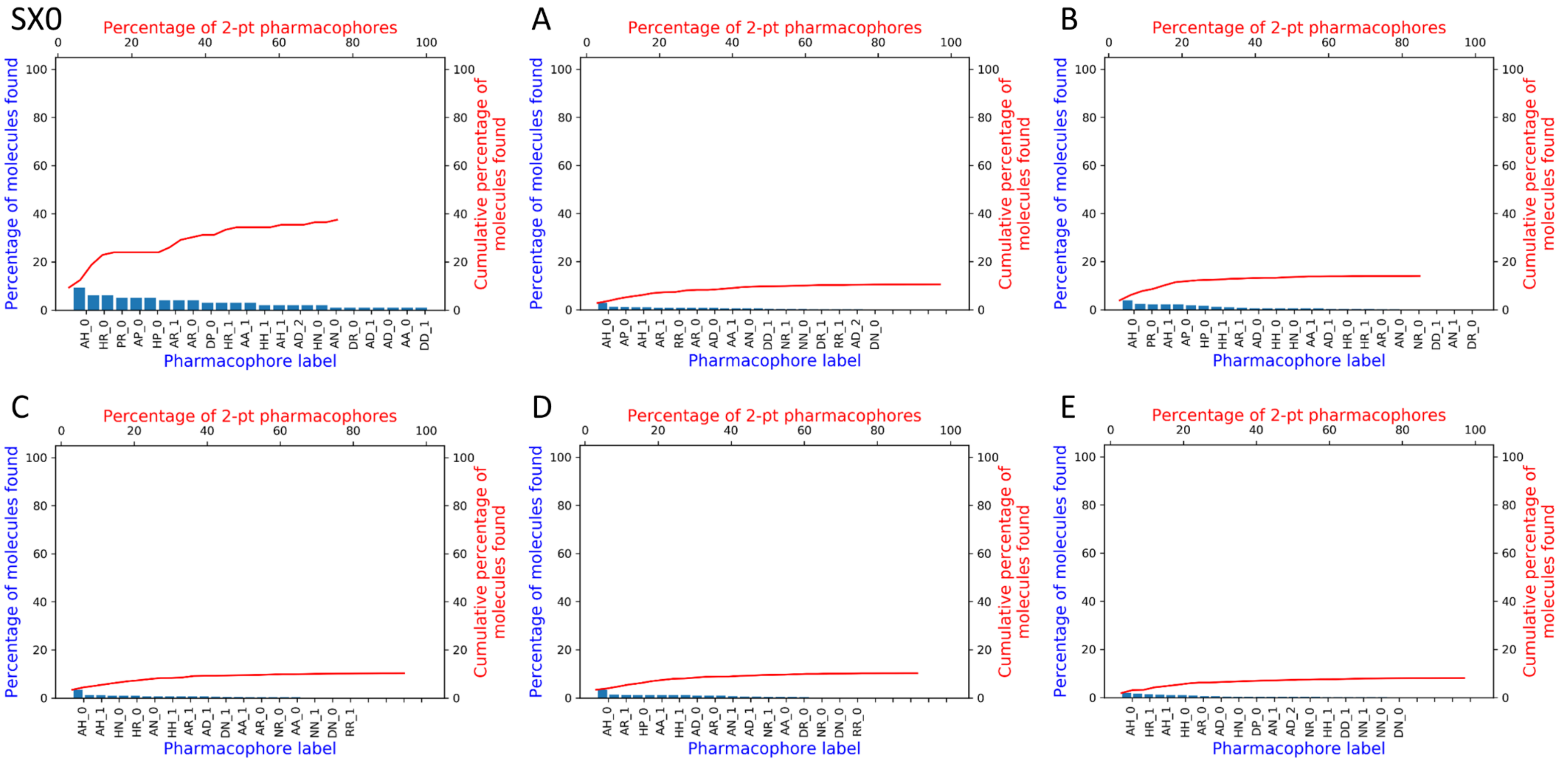


**Supplementary Figure 3. Percentage of molecules containing a specific pharmacophore (blue columns) and cumulative percentages of total molecules "found" (red line) by screening the complete set against the first x % of most populated 2-point pharmacophores (top x axis).** Only those pharmacophores are included that are present on their own right (i.e. not as submodels of a larger pharmacophore). The SpotXplorer pilot library (SX0) is compared to the top five most popular commercial fragment library providers (in random order, vendor names undisclosed due to business sensitivity) according to the poll conducted on the Practical Fragments blog.[27]

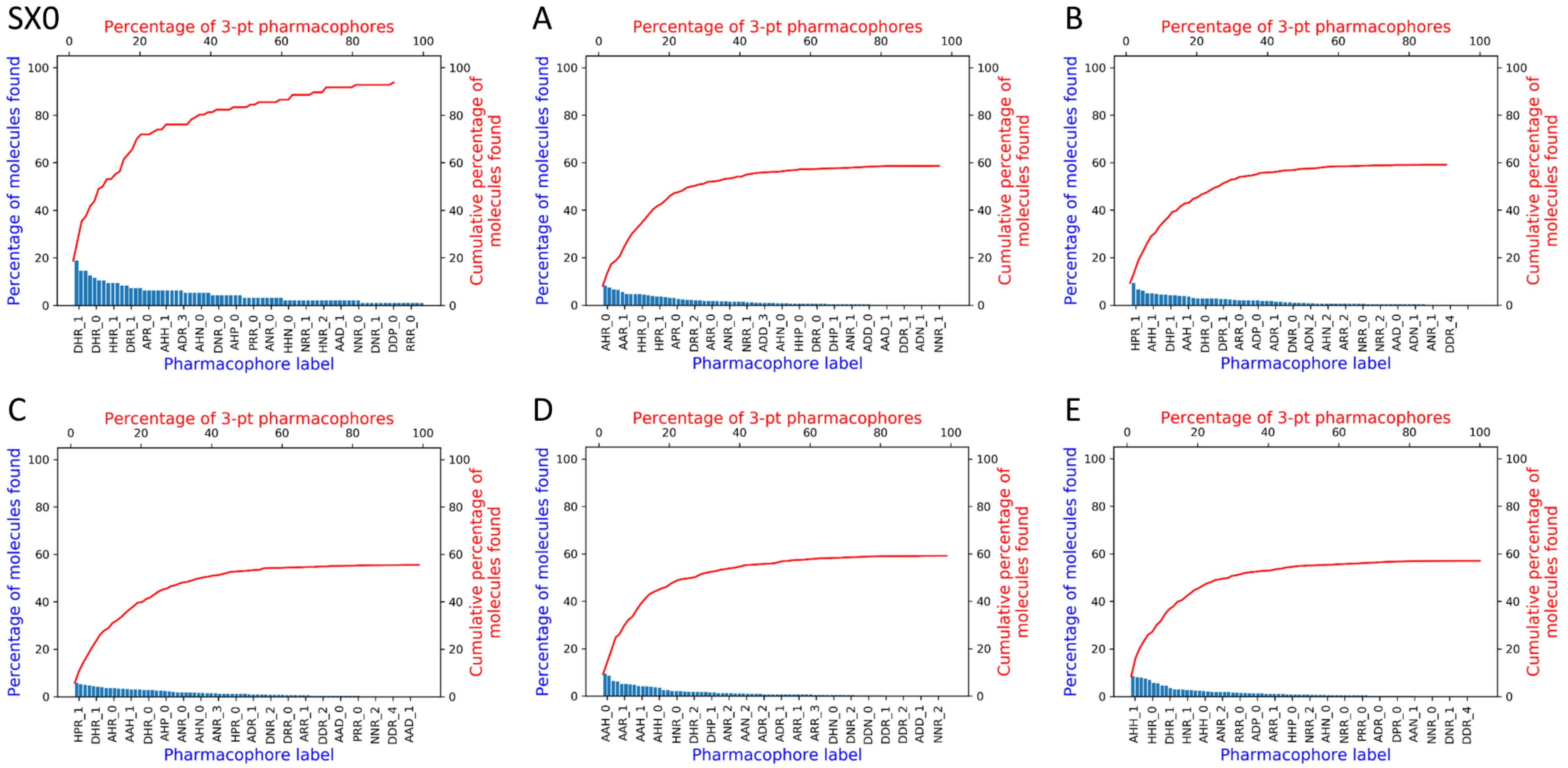


**Supplementary Figure 4. Percentage of molecules containing a specific pharmacophore (blue columns) and cumulative percentages of total molecules "found" (red line) by screening the complete set against the first x % of most populated 3-point pharmacophores (top x axis).** Only those pharmacophores are included that are present on their own right (i.e. not as submodels of a larger pharmacophore). The SpotXplorer pilot library (SX0) is compared to the top five most popular commercial fragment library providers (in random order, vendor names undisclosed due to business sensitivity) according to the poll conducted on the Practical Fragments blog.[27]

## 5. *In vitro* screening for selected GPCRs

*5.1 Cell culture and preparation of cell membranes for radioligand binding assays*

HEK293 cells (ATCC, cat. no. CRL-1573) with stable expression of human 5-$HT_{1A}$, 5-$HT_6$ and 5-$HT_{7b}$ receptors (prepared with the use of Lipofectamine 2000) were maintained at 37°C in a humidified atmosphere with 5% $CO_2$ and grown in Dulbecco's Modifier Eagle Medium containing 10% dialyzed fetal bovine serum and 500 mg/ml G418 sulfate. For membrane preparation, cells were subcultured in 150 $cm^2$ flasks, grown to 90% confluence, washed twice with prewarmed to 37°C phosphate buffered saline (PBS) and pelleted by centrifugation (200g) in PBS containing 0.1 mM EDTA and 1 mM dithiothreitol. Prior to membrane preparation, pellets were stored at –80°C.

*5.2 Radioligand binding assays*

Cell pellets were thawed and homogenized in 10 volumes of assay buffer using an Ultra Turrax tissue homogenizer and centrifuged twice at 35,000 g for 15 min at 4°C, with incubation for 15 min at 37°C in between. The composition of the assay buffers was as follows: for 5-$HT_{1A}$R: 50 mM Tris HCl, 0.1 mM EDTA, 4 mM $MgCl_2$, 10 μM pargyline and 0.1% ascorbate; for 5-$HT_6$R: 50 mM Tris HCl, 0.5 mM EDTA and 4 mM $MgCl_2$; for 5- $HT_{7b}$R: 50 mM Tris HCl, 4 mM $MgCl_2$, 10 μM pargyline and 0.1% ascorbate. All assays were incubated in a total volume of 200 μL in 96-well microtitre plates for 1 h at 37°C, except 5-$HT_{1A}$R which was incubated at room temperature. The process of equilibration was terminated by rapid filtration through Unifilter plates with a 96-well cell harvester and radioactivity retained on the filters was quantified on a Microbeta plate reader (PerkinElmer, USA). For displacement screening studies, the assay samples contained as radioligands (PerkinElmer, USA): 2.5 nM [$^3$H]-8-OH-DPAT (135.2 Ci/mmol) for 5-$HT_{1A}$R; 2 nM [$^3$H]-LSD (83.6 Ci/mmol) for 5-$HT_6$R or 0.8 nM [$^3$H]-5-CT (39.2 Ci/mmol) for 5-$HT_7$R. Non-specific binding was defined with 10 μM of 5-HT in 5-$HT_{1A}$R and 5-$HT_7$R binding experiments, whereas 10 μM of mianserin was used in 5-$HT_6$R assays, respectively. Each compound was tested at 10 μM concentration.

The % of inhibition (% inh) was calculated from the equation:

*% inh* = 100 x [1 – (*signal from compound @ 10 μM – non-specific binding*) / (*total [$^3$H]-radioligand binding – non-specific binding*)]

Results were expressed as means of two separate experiments, and % inhibition values below 0 or above 100 were corrected to 0 and 100%, respectively. Fragments that exhibited 50% or stronger inhibition were considered as actives.

## 6. Protease inhibitory assays

Spectrophotometric enzyme tests were performed in transparent microtiter plates in a final volume of 200 μL. The reaction rates in the absence and in the presence of the inhibitor were measured.

50 μL HBSA buffer – 10 mM Hepes buffer (HEPES, Sigma) with 0.1 % w/w BSA (bovine serum albumine), 150 mM NaCl, adjusted with 0.1 M NaOH to pH 7.5 –, 50 μL solution (4 % DMSO in water) of different inhibitors at 800 μM (or vehicle, in case of measurements without inhibitor) and 50 μL of protease solution (human thrombin, Sigma-Aldrich, 2 NIH E/mL or bovine factor Xa, Chromogenix, 2 nkat/mL) was pipetted into the microtiter plate. The plate

was incubated for 15 minutes at 25 °C and subsequently 50 µL chromogenic substrate – for thrombin: S-2238 (H-D-Phe-Pip-Arg-pNA•2HCl, Chromogenix), 160 µM; for bovine Factor Xa: S-2222 (Bz-IIe-Glu(γ-OR)-Gly-Arg-pNA•HCl, R:H/Me, Chromogenix), 400 µM – was added.

Final concentration of the inhibitors was 200 µM, DMSO 1 %, thrombin 0.5 NIH E/mL and substrate 40 µM, or factor Xa 0.5 nkat/mL and substrate 100 µM, respectively. The microtiter plate was put into the spectrophotometer (Biotek H4) and the increase of absorbance at 405 nm at 25°C was measured every 10 seconds. Change of absorbance form the initial, linear part of the curve was used to determine residual activity; screenings were carried out in duplicate in one independent experiment and $K_i$ values were determined in triplicate in two independent experiments. Values for $K_i$ were calculated according to Cheng and Prusoff based on the relation between reaction velocity equations in the absence and presence of inhibitor, using the relevant $K_m$ ($K_m$ = 2.6 µM for thrombin and $K_m$ = 164 µM for FXa).

**7. SETD2 Chemiluminescence assay**

The Chemiluminescence assay evaluates SETD2 specific tri-methylation activity towards histone H3K36. The kit consists of recombinant SETD2, S-adenosylmethionine and a specific H3K36me3 antibody and was performed according to the manufacturer's protocol (Catalog-No. 52060, https://bpsbioscience.com/setd2-chemiluminescent-assay-kit-52060). Values were blank corrected and normalized. Enzymatic activities were calculated relative to the positive control of the kit (full enzymatic activity) and to the known SET domain inhibitor Sinefungin[28] used as negative control (complete enzymatic activity inhibition). Compounds used were diluted in DMSO. $IC_{50}$ values were determined from at least five concentrations in duplicates, with serial dilutions starting from 1mM, using the Prism8 software (GraphPad, San Diego, CA, USA).

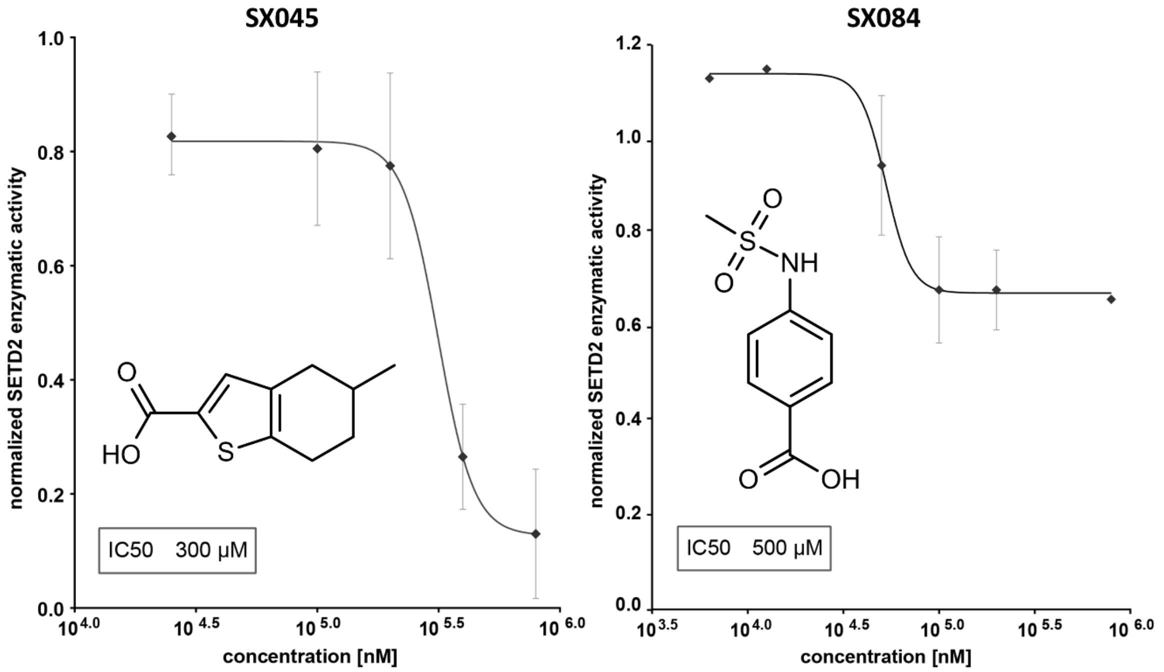


**Supplementary Figure 5. $IC_{50}$ curves of SX045 and SX084 against SETD2 in the Chemiluminescence assay.** Data are presented as mean values +/- SD, calculated from n = 2 biologically independent samples. Source data are provided in the Source Data file.

**8. Cell viability assay**

MOLM13 and MV4-11 leukemia cells (purchased from Deutsche Sammlung von Mikroorganismen und Zellkulturen GmbH, www.dsmz.de) were seeded in white 96-well plates and treated with SX045 in biological triplicates at indicated concentrations. Five days after treatment, cell viability was measured using the CellTiter-Glo Luminescent Cell Viability Assay (Promega, Madison, WI, USA) on a SPARK multimode microplate reader (Tecan Trading AG, Switzerland). $IC_{50}$ values were calculated using the Prism8 software (GraphPad, San Diego, CA, USA).

**9. X-ray fragment screening against COVID-19 targets**

Crystals of the 3CLPro and NSP3 macrodomain *apo* proteins were grown using the sitting drop vapor diffusion method at 20°C. NSP3 macrodomain *apo* crystals were grown in crystallization drops containing 150 nl of protein solution (47 mg/ml in 20 mM HEPES pH 8.0, 250 mM NaCl, and 2 mM DTT) plus 150 nl crystallization solution (100 mM CHES pH 9.5 and 30 % PEG3000).[29] For 3CLPro, crystallization drops contained 150 nl protein solution (5 mg/ml in 20 mM HEPES pH 7.5 and 50 mM NaCl), 300 nl crystallization solution (11% PEG 4 K, 5% DMSO, 0.1 M MES pH 6.7 ) and 50 nl seeds.[30] For both SARS-CoV-2 main protease (3CLPro) and NSP3 macrodomain, fragments were soaked into crystals by acoustic dispensing,[31] adding dissolved compound directly to the crystallisation drops using an ECHO liquid handler (final concentration 10% DMSO); drops were incubated for approximately 1-3 hours prior to mounting and flash freezing in liquid nitrogen.

Data were collected at the beamline I04-1 at 100K and automatically processed with Diamond Light Source's auto-processing pipelines using XDS[32] and either xia2,[33] autoPROC[34] or DIALS[35] with the default settings. Most SARS-CoV-2 main protease (3CLPro) data processed to a resolution of approximately 1.8 Å and NSP3 macrodomain to 1.1 Å. For both targets, data with resolution below 2.8 Å were excluded. Further analysis was performed with XChemExplorer,[36] electron density maps were generated with Dimple,[37] ligand-binding events were identified using PanDDA[38] (both the released version 0.2 and the pre-release development version https://github.com/ConorFWild/pandda). Ligands were modelled into PanDDA-calculated event maps using Coot[39], restraints were calculated with ACEDRG[40] or GRADE (version 1.2.19, Global Phasing Ltd., Cambridge, United Kingdom, 2010), and structures were refined with Refmac[41] and Buster (version 2.10.13, Cambridge, United Kingdom, 2017). Coordinates, structure factors and PanDDA event maps for the structures discussed are deposited in the Protein Data Bank (PDB IDs 5RHD, 5S4F, 5S4G, 5S4H, 5S4I and 5S4J). Data collection and refinement statistics are summarised in the Supplementary Data file.

In addition, the 3CLPro enzyme inhibition of SX013 was determined within the COVID Moonshot initiative (https://covid.postera.ai/covid), using the RapidFire SPE-MS assay set up at the Schofield group of the University of Oxford, Department of Chemistry. Briefly, a solution containing 20 nM Mpro, 20 mM HEPES, pH 7.5 and 50 mM NaCl, was added to the wells of an ECHO plate and incubated with SX013 for 15 min at room temperature. The reaction is initiated with 2.0 μM 3CLPro substrate (TSAVLQSGFRK, custom synthesized in Schofield group), quenched after 10 min with 10% formic acid and injected into an Agilent RapidFire

LC-MS system.[42] A dose-response curve was fitted to 11 datapoints in the range of 200 – 0.0034 μM SX013, with four replicates per datapoint.

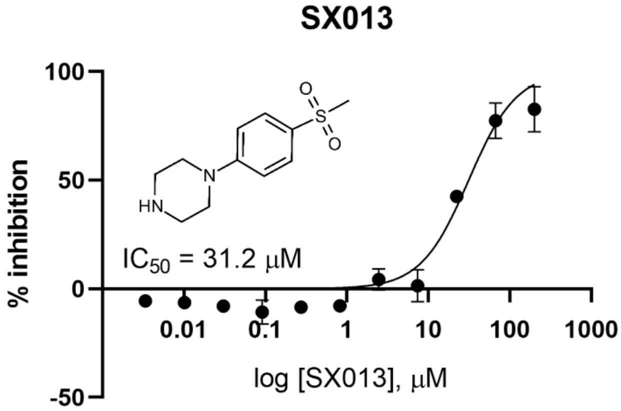


**Supplementary Figure 6. $IC_{50}$ curve of SX013 against SARS-COV-2 3CLPro in the RapidFire SPE-MS assay.** Data are presented as mean values +/- SD, calculated from n = 2 biologically independent samples, with 2 technical replicates each. Source data are provided in the Source Data file.

Inhibition of SpotXplorer0 fragment hits SX003, SX005, SX048, SX051 and SX054 against the SARS-CoV-2 NSP3 macrodomain was assessed by the displacement of an ADP-ribose-conjugated biotin peptide from the $His_6$-tagged macrodomain using HTRF with a $Eu^{3+}$-conjugated anti-$His_6$ antibody donor and streptavidin-conjugated acceptor.[29] Fragments were dispensed (1:1 serial dilution, 8 assay points) into white ProxiPlate-384 Plus (PerkinElmer) assay plates using an Echo 525 Liquid Handler (Labcyte), whereby SX003, SX005, SX051, SX054 were tested at a final assay concentration of 5 mM, SX048 at a final assay concentration of 12.5 mM. Binding assays were conducted in a final volume of 16 μl with 12.5 nM Nsp3 macrodomain, 400 nM peptide ARTK(Bio)QTARK(Aoa-RADP)S, 1:125 Streptavidin-XL665 (610SAXLB, CisBio), 1:20000 Anti-$His_6$-$Eu^{3+}$ cryptate (AD0111, PerkinElmer) in assay buffer (25 mM HEPES pH7.0, 20 mM NaCl, 0.05% BSA, 0.05% Tween20). Assay reagents were dispensed into plates using a Multidrop combi (Thermo Scientific) and incubated at room temperature for 1 h. Fluorescence was measured using a PHERAstar microplate reader (BMG) using the HTRF module with dual emission protocol (A = excitation of 320 nm, emission of 665 nm, and B = excitation of 320 nm, emission of 620 nm). Raw data were processed to give an HTRF ratio (channel A/B × 10,000), which was used to generate $IC_{50}$ curves by nonlinear regression using GraphPad Prism v8 (GraphPad Software, CA, USA).

These measurements revealed that SX005 and SX048 inhibited NSP3 with $IC_{50}$ values of 465 μM and 5.5 mM, respectively.

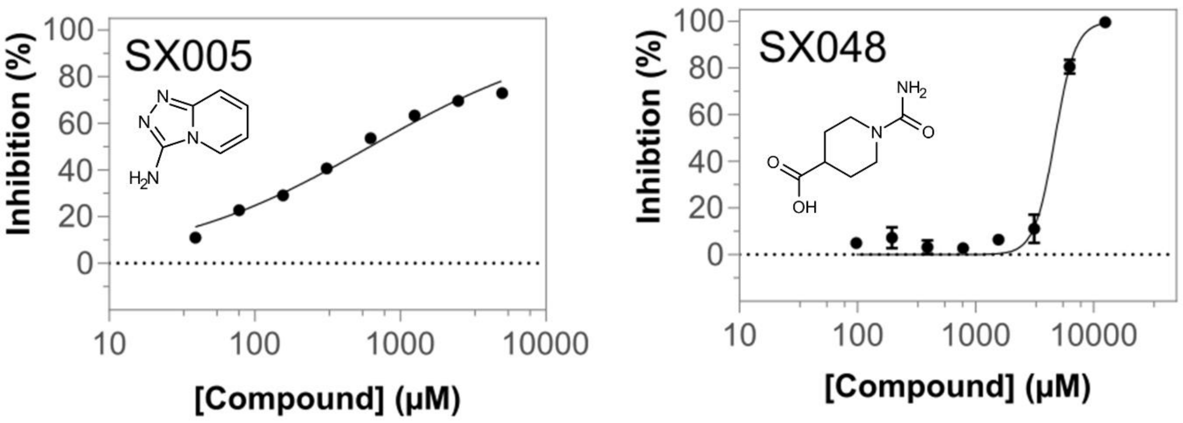


**Supplementary Figure 7. $IC_{50}$ curves of SX005 and SX048 against SARS-COV-2 NSP3 in the HTRF assay.** Data are presented as mean values +/- SD, calculated from n = 2 biologically independent samples. Source data are provided in the Source Data file.

**10. *In vitro* SARS-CoV-2 antiviral activity screen**

Vero E6 cells (purchased from the European Collection of Authenticated Cell Cultures) were seeded the day before the experiment in a 96-well plate. On the following day, cells were treated with SX003, SX005, SX013, SX048, SX051 or SX054 (1 mM to 10 μM). Immediately after treatment, cells were infected with SARS-CoV-2 (hCoV-19/Hungary/SRC_isolate_2/2020, GISAID ID: EPI_ISL_483637) at MOI: 0.01. Cells were incubated for 30 min at 37°C, then the supernatant was replaced with fresh maintenance media supplemented with the compounds at the appropriate concentration. 48 hours post infection nucleic acid extraction was made from the supernatant (New England BioLabs® Inc. Monarch® Total RNA Miniprep Kit). Viral copy numbers were determined using SARS-CoV-2 RdRp gene specific primers and probe (see Supplementary Table 2) and droplet digital PCR (Bio-Rad Laboratories Inc. QX200 Droplet Digital PCR System). $EC_{50}$ values were determined using non-linear regression analysis (GraphPad Prism 8). The $EC_{50}$ curves for SX013 and SX051 are shown in the main text (Figure 4), while those of SX005 and SX048 are shown here.

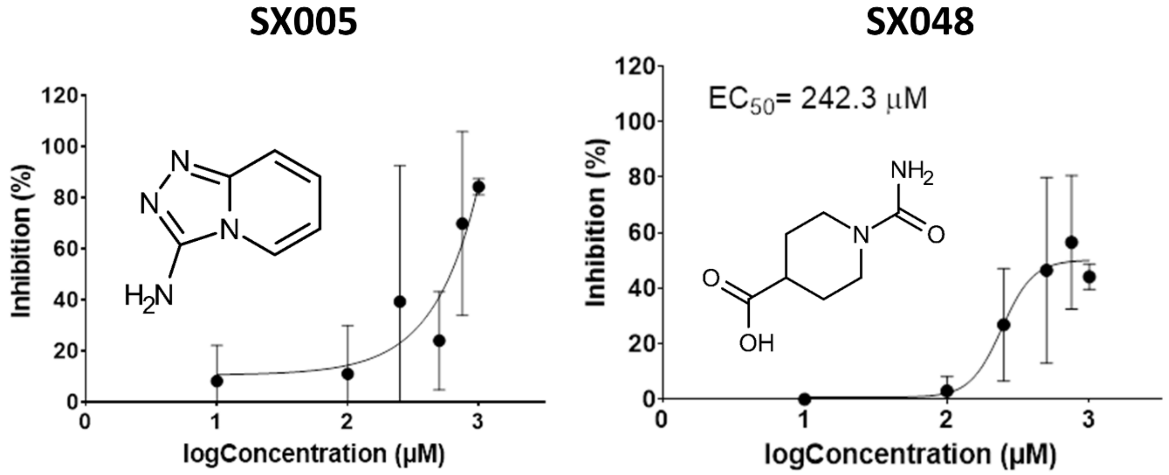


**Supplementary Figure 8. $EC_{50}$ curves of SX005 and SX048 against SARS-COV-2-infected Vero E6 cells.** Data are presented as mean values +/- SD, calculated from n = 3 biologically independent samples. Source data are provided in the Source Data file.

**Supplementary Table 2. Names and sequences of the applied primers.**

| **Name** | **Sequence** |
|---|---|
| ***In vitro* screening for selected GPCRs** | |
| HT1for | GAT CGA GGT GCA CCG AGT GG |
| HT1rev | CCC ATG ATG ATG CCC AGC GT |
| HT6for | GGG CAT GTT CTT TGT GAC CT |
| HT6rev | AGT AAC CCA GCC ATG TGA GG |
| HT7for | TCA GCC AGG ACT TTG GCT AT |
| HT7rev | TGT GTT TGG CAG CAC TCT TC |
| ***In vitro* SARS-CoV-2 antiviral activity screen** | |
| IDT 2019-nCoV Charité/Berlin forward primer | GTGARATGGTCATGTGTGGCGG |
| IDT 2019-nCoV Charité/Berlin reverse primer | CARATGTTAAASACACTATTAGCATA |
| IDT 2019-nCoV Charité/Berlin probe | FAM-CAGGTGGAACCTCATCAGGAGATGC-BBQ |

## 11. Supplementary references


1. Murray, C. W. & Rees, D. C. Opportunity Knocks: Organic Chemistry for Fragment-Based Drug Discovery (FBDD). *Angew. Chemie Int. Ed.* **55**, 488–492 (2016).

2. Berthold, M. R. *et al.* {KNIME}: The {K}onstanz {I}nformation {M}iner. in *Studies in Classification, Data Analysis, and Knowledge Organization (GfKL 2007)* (Springer, 2007).

3. O'Boyle, N. M. & Hutchison, G. R. Cinfony – combining Open Source cheminformatics toolkits behind a common interface. *Chem. Cent. J.* **2**, 24 (2008).

4. Sastry, G. M., Adzhigirey, M., Day, T., Annabhimoju, R. & Sherman, W. Protein and ligand preparation: parameters, protocols, and influence on virtual screening enrichments. *J. Comput. Aided. Mol. Des.* **27**, 221–34 (2013).

5. Friesner, R. A. *et al.* Extra Precision Glide: Docking and Scoring Incorporating a Model of Hydrophobic Enclosure for Protein−Ligand Complexes. *J. Med. Chem.* **49**, 6177–6196 (2006).

6. Salam, N. K., Nuti, R. & Sherman, W. Novel Method for Generating Structure-Based Pharmacophores Using Energetic Analysis. *J. Chem. Inf. Model.* **49**, 2356–2368 (2009).

7. Brenke, R. *et al.* Fragment-based identification of druggable 'hot spots' of proteins using Fourier domain correlation techniques. *Bioinformatics* **25**, 621–627 (2009).

8. Kozakov, D. *et al.* The FTMap family of web servers for determining and characterizing ligand-binding hot spots of proteins. *Nat. Protoc.* **10**, 733–755 (2015).

9. Mattos, C. & Ringe, D. Locating and Characterizing Binding Sites on Proteins. *Nat. Biotechnol.* **14**, 595–599 (1996).

10. Allen, K. N. *et al.* An experimental approach to mapping the binding surfaces of crystalline proteins. *J. Phys. Chem.* **100**, 2605–2611 (1996).

11. Hajduk, P. J., Meadows, R. P. & Fesik, S. W. NMR-based screening in drug discovery. *Quarterly Reviews of Biophysics* vol. 32 211–240 (1999).

12. Brooks, B. R. *et al.* CHARMM: A program for macromolecular energy, minimization, and dynamics calculations. *J. Comput. Chem.* **4**, 187–217 (1983).

13. Schaefer, M. & Karplus, M. A comprehensive analytical treatment of continuum electrostatics. *J. Phys. Chem.* **100**, 1578–1599 (1996).

14. Kozakov, D. *et al.* New Frontiers in Druggability. *Journal of Medicinal Chemistry* vol. 58 9063–9088 (2015).

15. Hall, D. R., Kozakov, D., Whitty, A. & Vajda, S. Lessons from Hot Spot Analysis for Fragment-Based Drug Discovery. *Trends Pharmacol. Sci.* **36**, 724–736 (2015).

16. Hall, D. R., Ngan, C. H., Zerbe, B. S., Kozakov, D. & Vajda, S. Hot spot analysis for driving the development of hits into leads in fragment-based drug discovery. *J. Chem. Inf. Model.* **52**, 199–209 (2012).

17. Jones, E., Oliphant, T., Peterson, P. & others. {SciPy}: Open source scientific tools for {Python}. http://www.scipy.org/.

18. Allen, W. J. & Rizzo, R. C. Implementation of the Hungarian Algorithm to Account for Ligand Symmetry and Similarity in Structure-Based Design. *J. Chem. Inf. Model.* **54**, 518–529 (2014).

19. Ferenczy, G. G. & Keserű, G. M. Thermodynamics of Fragment Binding. *J. Chem. Inf. Model.* **52**, 1039–1045 (2012).

20. Dixon, S. L., Smondyrev, A. M. & Rao, S. N. PHASE: A novel approach to pharmacophore modeling and 3D database searching. *Chemical Biology and Drug Design* vol. 67 370–372 (2006).

21. Dixon, S. L. *et al.* PHASE: a new engine for pharmacophore perception, 3D QSAR model development, and 3D database screening: 1. Methodology and preliminary results. *J. Comput. Aided. Mol. Des.* **20**, 647–671 (2006).

22. Baell, J. B. & Holloway, G. A. New substructure filters for removal of pan assay interference compounds (PAINS) from screening libraries and for their exclusion in bioassays. *J. Med. Chem.* **53**, 2719–2740 (2010).

23. Shelley, J. C. *et al.* Epik: a software program for pK a prediction and protonation state generation for drug-like molecules. *J. Comput. Aided. Mol. Des.* **21**, 681–691 (2007).

24. Greenwood, J. R., Calkins, D., Sullivan, A. P. & Shelley, J. C. Towards the comprehensive, rapid, and accurate prediction of the favorable tautomeric states of drug-like molecules in aqueous solution. *J. Comput. Aided. Mol. Des.* **24**, 591–604 (2010).

25. Schrödinger Release 2017-4: Macromodel, Schrödinger, LLC, New York, NY, 2017.

26. Ashton, M. *et al.* Identification Of Diverse Database Subsets Using Property-Based And Fragment-Based Molecular Descriptions. *Quant. Struct. Relationships* **21**, 598–604 (2002).

27. Erlanson, D. Poll results: library vendors. http://practicalfragments.blogspot.com/2018/12/poll-results-library-vendors.html (2018).

28. Zheng, W. *et al.* Sinefungin derivatives as inhibitors and structure probes of protein lysine methyltransferase SETD2. *J. Am. Chem. Soc.* **134**, 18004–18014 (2012).

29. Schuller, M. *et al.* Fragment binding to the Nsp3 macrodomain of SARS-CoV-2 identified through crystallographic screening and computational docking. *Sci. Adv.* **7**, eabf8711 (2021).

30. Douangamath, A. *et al.* Crystallographic and electrophilic fragment screening of the SARS-CoV-2 main protease. *Nat. Commun.* **11**, 5047 (2020).

31. Collins, P. M. *et al.* Gentle, fast and effective crystal soaking by acoustic dispensing. *Acta Crystallogr. Sect. D Biol. Crystallogr.* **73**, 246–255 (2017).

32. Kabsch, W. *et al.* XDS. *Acta Crystallogr. Sect. D Biol. Crystallogr.* **66**, 125–132 (2010).

33. Winter, G., Lobley, C. M. C. & Prince, S. M. Decision making in xia2. *Acta Crystallogr. Sect. D Biol. Crystallogr.* **69**, 1260–1273 (2013).

34. Vonrhein, C. *et al.* Data processing and analysis with the autoPROC toolbox. *Acta Crystallogr. Sect. D Biol. Crystallogr.* **67**, 293–302 (2011).

35. Winter, G. *et al.* DIALS: Implementation and evaluation of a new integration package. *Acta Crystallogr. Sect. D Struct. Biol.* **74**, 85–97 (2018).

36. Krojer, T. *et al.* The XChemExplorer graphical workflow tool for routine or large-scale protein-ligand structure determination. *Acta Crystallogr. Sect. D Biol. Crystallogr.* **73**, 267–278 (2017).

37. Wojdyr, M., Keegan, R., Winter, G. & Ashton, A. DIMPLE - a pipeline for the rapid generation of difference maps from protein crystals with putatively bound ligands. *Acta Crystallogr. Sect. A Found. Adv.* **69**, s299 (2013).

38. Pearce, N. M. *et al.* A multi-crystal method for extracting obscured crystallographic states from conventionally uninterpretable electron density. *Nat. Commun.* **8**, 15123 (2017).

39. Emsley, P., Lohkamp, B., Scott, W. G. & Cowtan, K. Features and development of Coot. *Acta Crystallogr. Sect. D Biol. Crystallogr.* **66**, 486–501 (2010).

40. Long, F. *et al.* AceDRG: A stereochemical description generator for ligands. *Acta Crystallogr. Sect. D Struct. Biol.* **73**, 112–122 (2017).

41. Murshudov, G. N. *et al.* REFMAC5 for the refinement of macromolecular crystal structures. *Acta Crystallogr. Sect. D Biol. Crystallogr.* **67**, 355–367 (2011).

42. Malla, T. R. *et al.* Mass spectrometry reveals potential of β-lactams as SARS-CoV-2 M pro inhibitors. *Chem. Commun.* **57**, 1430–1433 (2021).
