# Supplementary material for: Exploring protein hotspots by optimized fragment pharmacophores
Source: Nat Commun. 2021 May 27;12:3201. doi: 10.1038/s41467-021-23443-y (PMC8159961; doi:10.1038/s41467-021-23443-y)
Supplement: Supplementary file 3 — Description of Additional Supplementary Files [file 41467_2021_23443_MOESM3_ESM.docx]

Description of Additional Supplementary Files

Title: Supplementary Data 1

Description: List of ligands that were filtered out by manual curation. List of PDB entries after preparation and filtering steps. List of compounds in SpotXplorer 0 with SMILES, vendor Ids, experimental hit status and pharmacophore fingerprints. 2D structures of the fragments as images, solubility data. Primary screening data of SpotXplorer 0 against the GPCR panel (% inhibition values). Screening data of SpotXplorer 0 against the protease panel and SETD2 (Ki and IC50 values). PDB IDs and crystallographic information of the six co-crystallized fragment-protein complexes.
