# Supplementary material for: Exploring protein hotspots by optimized fragment pharmacophores
Source: Nat Commun. 2021 May 27;12:3201. doi: 10.1038/s41467-021-23443-y (PMC8159961; doi:10.1038/s41467-021-23443-y)
Supplement: Supplementary file 5 — Reporting Summary [file 41467_2021_23443_MOESM5_ESM.pdf]

nature research

Corresponding author(s): György M. Keserű

Last updated by author(s): Apr 19, 2021

Reporting Summary

Nature Research wishes to improve the reproducibility of the work that we publish. This form provides structure for consistency and transparency in reporting. For further information on Nature Research policies, see our Editorial Policies and the Editorial Policy Checklist.

Statistics

For all statistical analyses, confirm that the following items are present in the figure legend, table legend, main text, or Methods section.

n/a	Confirmed	
☐	☒	The exact sample size (n) for each experimental group/condition, given as a discrete number and unit of measurement
☐	☒	A statement on whether measurements were taken from distinct samples or whether the same sample was measured repeatedly
☒	☐	The statistical test(s) used AND whether they are one- or two-sided<br>*Only common tests should be described solely by name; describe more complex techniques in the Methods section.*
☒	☐	A description of all covariates tested
☒	☐	A description of any assumptions or corrections, such as tests of normality and adjustment for multiple comparisons
☐	☒	A full description of the statistical parameters including central tendency (e.g. means) or other basic estimates (e.g. regression coefficient) AND variation (e.g. standard deviation) or associated estimates of uncertainty (e.g. confidence intervals)
☒	☐	For null hypothesis testing, the test statistic (e.g. F, t, r) with confidence intervals, effect sizes, degrees of freedom and P value noted<br>*Give P values as exact values whenever suitable.*
☒	☐	For Bayesian analysis, information on the choice of priors and Markov chain Monte Carlo settings
☒	☐	For hierarchical and complex designs, identification of the appropriate level for tests and full reporting of outcomes
☒	☐	Estimates of effect sizes (e.g. Cohen's d, Pearson's r), indicating how they were calculated

Our web collection on statistics for biologists contains articles on many of the points above.

Software and code

Policy information about availability of computer code

Data collection	To assemble the non-redundant set of binding pharmacophores, protein-fragment complex structures were downloaded from the Protein Data Bank with the batch download tool of PDB. A SPARK multimode plate reader (Tecan Trading AG, Switzerland) was used to measure the SETD2 chemiluminescence assay and the cell viability assay. A Microbeta plate reader was used for the radioligand binding assay. For the SARS-CoV-2 antiviral activity screen, viral copy numbers were determined with a Bio-Rad Laboratories Inc. QX200 Droplet Digital PCR system.
Data analysis	Pharmacophore models were extracted from the PDB structures with the ePharmacophore module of the Schrödinger software suite (version 2017-4), and clustered with scipy (version 1.0.1). Ligand preparation, conformer generation and pharmacophore screening was carried out with the Epik, Macromodel and Phase modules of the Schrödinger software suite, respectively (version 2017-4). Binding hot-spots were detected with FTMap (http://ftmap.bu.edu). Statistical analysis of assay data was performed with GraphPad Prism software 8.0.

For manuscripts utilizing custom algorithms or software that are central to the research but not yet described in published literature, software must be made available to editors and reviewers. We strongly encourage code deposition in a community repository (e.g. GitHub). See the Nature Research guidelines for submitting code & software for further information.

Data

Policy information about availability of data

All manuscripts must include a data availability statement. This statement should provide the following information, where applicable:

- Accession codes, unique identifiers, or web links for publicly available datasets
- A list of figures that have associated raw data
- A description of any restrictions on data availability

Structure data that support the findings of this study have been deposited in the PDB database (https://rcsb.org), with the accession codes 5RHD [http://doi.org/10.2210/pdb5RHD/pdb], 5S4F [http://doi.org/10.2210/pdb5S4F/pdb], 5S4G [http://doi.org/10.2210/pdb5S4G/pdb], 5S4H [http://doi.org/10.2210/

pdb5S4H/pdb], 5S4I [http://doi.org/10.2210/pdb5S4I/pdb] and 5S4J [http://doi.org/10.2210/pdb5S4J/pdb]. Data generated during the computational and experimental screening of the described fragment library are reported in Supplementary Data 1. Structures for assembling the set of non-redundant pharmacophores were downloaded from the Protein Data Bank (https://www.rcsb.org/). GPCR and protease ligands and bioactivity data were downloaded from the ChEMBL database (https://www.ebi.ac.uk/chembl/). Source data are provided with this paper.

Field-specific reporting

Please select the one below that is the best fit for your research. If you are not sure, read the appropriate sections before making your selection.

[x] Life sciences   [ ] Behavioural & social sciences   [ ] Ecological, evolutionary & environmental sciences

For a reference copy of the document with all sections, see nature.com/documents/nr-reporting-summary-flat.pdf

Life sciences study design

All studies must disclose on these points even when the disclosure is negative.

Sample size

The radioligand binding assays (for GPCR targets), protease inhibitory assays, as well as the SARS-CoV-2 3CLPro and NSP3 inhibitory assays were performed in biological duplicates, with at least two technical replicates. The SETD2 chemiluminescence assay as well as the follow-up cell viability assay in MOLM-13 and MV4-11 cells was performed in triplicates. In the cellular SARS-CoV-2 in vitro inhibition assay 3 biological replicates of each concentration were used to detemine the EC50 value of the compounds. Total RNA extraction was made from the whole supernatant of each sample.

Sample sizes were determined considering the cost vs. robustness balance of the experiments. Chosen sample sizes were sufficient to produce IC50 curves with little error, and were similar to earlier experiments, in which sufficient statistical power was achieved (GPCRs: Vanda et al. J Med Chem 2021; proteases: Ilaš et al. J Med Chem 2008; SETD2: Skucha et al., Nat Comm. 2018; SARS-CoV-2 3CLPro: Douangamath et al., Nat. Comm. 2020; NSP3: Schuller et al. Sci Adv. 2021).

Data exclusions

No data were excluded from this study.

Replication

The radioligand binding assays (for GPCR targets), protease inhibitory assays, as well as the SARS-CoV-2 3CLPro and NSP3 inhibitory assays were performed in biological duplicates, with at least two technical replicates. The SETD2 chemiluminescence assay and the cell viability assay were performed in two biological replicates. In the SARS-CoV-2 in vitro inhibition assay 3 biological replicates of each concentration were used to detemine the EC50 value of the compounds.

All attempts at replication were successful.

Randomization

As we do not observe any type of bias in microplates (plate bias, within-plate spatial bias, across-plate bias), randomization was not performed directly during testing. Nonetheless, the order of the compounds on the plate can be considered random, as they are not grouped or arranged by any specific structural feature (they are grouped by vendors).

Blinding

No structures were disclosed to the experimenters until the final results of the assays were determined.

Reporting for specific materials, systems and methods

We require information from authors about some types of materials, experimental systems and methods used in many studies. Here, indicate whether each material, system or method listed is relevant to your study. If you are not sure if a list item applies to your research, read the appropriate section before selecting a response.

Materials & experimental systems

n/a	Involved in the study	
[x]	[ ]	Antibodies
[ ]	[x]	Eukaryotic cell lines
[x]	[ ]	Palaeontology and archaeology
[x]	[ ]	Animals and other organisms
[x]	[ ]	Human research participants
[x]	[ ]	Clinical data
[x]	[ ]	Dual use research of concern

Methods

n/a	Involved in the study	
[x]	[ ]	ChIP-seq
[x]	[ ]	Flow cytometry
[x]	[ ]	MRI-based neuroimaging

April 2020

Eukaryotic cell lines

Policy information about cell lines

Cell line source(s)	MOLM-13 and MV4-11 cells were obtained from DSMZ (Deutsche Sammlung von Mikroorganismen und Zellkulturen GmbH (DSMZ, www.dsmz.de). HEK-293 cell line was purchased from ATCC (American Type Culture Collection, cat. no. CRL-1573). Vero E6 cell line was purchased from ECACC (European Collection of Authenticated Cell Cultures).
Authentication	Cell line authetication was provided by the suppliers, using STR profiling (HEK293) and DNA fingerprinting (MOLM-13, MV4-11, Vero E6), respectively.
Mycoplasma contamination	MOLM-13 and MV4-11: Mycoplasma contamination was routinely tested (Lonza Kit, #LT07-318, every second month). Only mycoplasma free cells were used in the experiments. HEK-293: All cell lines were tested for mycoplasma contamination and the obtained results were negative. Vero E6 cells were negative for multiple mycoplasma species. The protocol was derived from Cord C. Uphoff and Hans G. Drexler. (References: doi: 10.1007/978-1-61779-080-5_8 doi: 10.1290/1071-2690(2002)038<0079:CPAFDO>2.0.CO;2).
Commonly misidentified lines (See ICLAC register)	No commonly misidentified cell lines were used.
